# Differences in salinity tolerance of genetically distinct *Phragmites australis* clones

**DOI:** 10.1093/aobpla/plt019

**Published:** 2013-03-11

**Authors:** Luciana Achenbach, Franziska Eller, Loc Xuan Nguyen, Hans Brix

**Affiliations:** Department of Bioscience, Plant Biology, Aarhus University, Ole Worms Alle 1, DK-8000 Aarhus C, Denmark

**Keywords:** Common reed, ecophysiology, geographic range, ion concentration, ploidy level, salt-stress tolerance

## Abstract

Different clones of the wetland grass *Phragmites australis* differ in their morphology and physiology, and hence in their ability to cope with environmental stress. We analysed the responses of 15 *P. australis* clones with distinct ploidy levels (PLs) (4*n*, 6*n*, 8*n*, 10*n*, 12*n*) and geographic origins (Romania, Russia, Japan, Czech Republic, Australia) to step-wise increased salinity (8, 16, 24, 32, 40, 56 and 72 ppt). Shoot elongation rate, photosynthesis and plant part-specific ion accumulation were studied in order to assess if traits associated with salinity tolerance can be related to the genetic background and the geographic origin of the clones. Salt stress affected all clones, but at different rates. The maximum height was reduced from 1860 mm in control plants to 660 mm at 40 ppt salinity. The shoot elongation rate of salt-exposed plants varied significantly between clones until 40 ppt salinity. The light-saturated photosynthesis rate (*P*_max_) was stimulated by a salinity of 8 ppt, but decreased significantly at higher salinities. The stomatal conductance (*g*_s_) and the transpiration rate (*E*) decreased with increasing salinity. Only three clones survived at 72 ppt salinity, although their rates of photosynthesis were strongly inhibited. The roots and basal leaves of the salt-exposed plants accumulated high concentrations of water-extractable Na^+^ (1646 and 1004 µmol g^−1^ dry mass (DM), respectively) and Cl^−^ (1876 and 1400 µmol g^−1^ DM, respectively). The concentrations of water-extractable Mg^2+^ and Ca^2+^ were reduced in salt-exposed plants compared with controls. The variation of all the measured parameters was higher among clones than among PLs. We conclude that the salinity tolerance of distinct *P. australis* clones varies widely and can be partially attributed to their longitudinal geographic origin, but not to PL. Further investigation will help in improving the understanding of this species' salt tolerance mechanisms and their connection to genetic factors.

## Abstract (precis)

The common reed *(Phragmites australis)* is a clonal wetland grass with high genetic variability. Clone-specific differences are reflected in morphological and physiological traits, and hence in the ability to cope with environmental stress. The responses to progressively increasing salinity of fifteen distinct *Phragmites australis* clones reveal genotype-related strategies of salt avoidance and exclusion. The salinity-induced inhibition in shoot elongation rate and photosynthesis varies widely between clones. The differences can be partially attributed to their geographic range, but not correlated to ploidy level. Thus, the genetic background is a major factor influencing the salinity tolerance of distinct *Phragmites australis* clones.

## Introduction

The common reed (*Phragmites australis* (Cav.) Trin ex Steud.) is a rhizomatous perennial grass with perhaps the largest geographical distribution of any flowering plant in the world (Brix 1999; Clevering and Lissner 1999). It is found in the littoral zones of lakes, along rivers and canals, and in shallow freshwater swamps, where it forms dense, nearly monospecific stands. Furthermore, *P. australis* grows in salt marshes and on salinized soils (Mauchamp and Mesleard 2001). The reported salt tolerance of *P. australis* differs between studies. Gorai *et al.* (2011) reported that *P. australis* grew optimally at salinities up to 100 mM (5.84 ppt), but showed significant signs of stress beyond this concentration. Reduced growth, as well as lowered photosynthesis and stomatal conductance, are some of the found effects of salinity stress of *P. australis* (Hanganu *et al.* 1999; Lissner *et al.* 1999*a*, *b*). Pagter *et al.* (2009) found osmotic and ion-specific effects of salt-stress on growth, gas- and water exchange, osmolality of leaf sap and tissue mineral composition.

The high genetic variability of *P. australis* has been reported by several studies (Clevering 1999; Hansen *et al.* 2007; Lambertini *et al.* 2008; Achenbach *et al.* 2012). The phylogeny of *P. australis* is complex (Lambertini *et al.* 2006); several ploidy levels (PLs) (2*n*= 3x, 4x, 6x, 8x, 10x, 12x) and phylogeographic clusters have been identified. The morphological, physiological and biochemical differences are significant between genotypes, yet they are not necessarily related to geographic origin or PL (Saltonstall 2007; Achenbach *et al.* 2012). Other studies have found correlations between PL and morphology, with octoploids often reported as taller and with thicker shoots and leaves than tetraploids (Pauca-Comanescu *et al.* 1999). Genotype-related differences in phenotypic plasticity (Eller and Brix 2012), as well as in physiological traits, such as the light-saturated rate of photosynthesis (*P*_max_) (Hansen *et al.* 2007), stomatal conductance (*g*_s_) and transpiration rates (*E*) (Achenbach *et al.* 2012), have also been reported.

Although differences in morphology and physiological traits have been described, the response of distinct *P. australis* genotypes to salinity stress and their range of tolerance remain to be elucidated. Differences in salinity tolerance between genotypes have been reported in the Danube Delta, where the growth of octoploids was found to be less affected by saline environments than the growth of hexa- or tetraploids (Pauca-Comanescu *et al.* 1999). A gradient of salt-tolerant genotypes was also observed in the Yellow River Delta (Gao *et al.* 2012).

The present paper provides details of the morphological (shoot height and elongation rate) and physiological (*P*_max_, *g*_s_, *E*) responses of distinct *P. australis* clones as affected by salinity. Additionally, photosynthetic pigments and water-extractable ion concentrations were analysed and variations between salt-exposed and control plants are reported.

Seven European clones and eight Asiatic/Australian clones with different PLs were exposed to progressively increasing salinities. The chosen clones have their native range at similar latitudes; thus, the effect of longitude is considered for the first time, complementing earlier studies that have evaluated the effect of latitudinal gradients on *P. australis* (Lissner *et al.* 1997; Clevering *et al.* 2001; Lessmann *et al.* 2001; Karunaratne *et al.* 2003; Bastlova *et al*. 2004).

We hypothesize that salinity tolerance is related to PL, as well as to the geographic distribution range (GR) of the clones, as clones occurring in Asia–Australia are predominantly octoploid, whereas clones occurring in Europe are predominantly tetraploid (Clevering and Lissner 1999). The study aimed at assessing the variability in salinity tolerance between the clones and the possible relation to their genetic background.

## Methods

### Plant material

The 15 clones used in this study were chosen from a large collection of live *P. australis* clones, kept in a common outdoor environment under similar conditions in terms of soil, water, nutrition and climate environment at Aarhus University, Denmark (56°13′N; 10°07′E), for at least 6 years prior to this study. Clones with distinct PLs (2*n*= 4x, 6x, 8x, 10x and 12x) and different geographical distribution ranges (Europe and Asia/Australia) were selected for the study (Table 1). Two clones of each PL were chosen to represent the European versus the Asiatic/Australian longitudinal variation. However, dodecaploids (12x), which only occur in Europe (Clevering and Lissner 1999), were only represented by a single clone, and tetraploids (4x) only by a single clone in Asia/Australia. The Asiatic/Australian group comprised three octoploid (8x) clones (from Japan, Australia and Sakhalin Island in Russia). Decaploids (10x), which occur only in Asia/Australia (Clevering and Lissner 1999), were represented by clones from Russia and New South Wales (Australia). Phylogenetically, the clones belonged to the ‘*P. australis* core group’, which is a large and mostly tetraploid (2*n*= 4x) group dominating in Europe and in North America, and to the ‘*P. australis* Australia–East Asia group’, which comprises mainly octo- and decaploid clones from Australia and tropical and temperate East Asia (Lambertini *et al.* 2006).

**Table 1:** List of *P. australis* clones used in this study, their origin, PL and phylogeographic relationships. Sample labels are as in Lambertini *et al*. (2006), but the prefix ‘Pa’, standing for *P. australis*, has been replaced by ‘E’ and ‘A’, indicating the geographic distribution of the clones in Europe (E) or in Asia/Australia (A).

Sample label	Origin	Ploidy level	Phylogeographic relationships^a^
E646RO4x	Romania, Lake Razim	4x	*P. australis* core group
E620CZ4x	Czech Republic, Rozmberk	4x	*P. australis* core group
E625RO6x	Romania, Lake Oborny	6x	*P. australis* core group
E656RO6x	Romania, Lake Razim	6x	*P. australis* core group
E624RO8x	Romania, Lake Obretinu	8x	*P. australis* core group
E666CZ8x	Czech Republic	8x	*P. australis* core group
E660RO12x	Romania, Lake Razim	12x	*P. australis* core group
A205RU4x	Russia, Sakhalin, Novikovo	4x	*P. australis* core group
A139RU6x	Russia, Sakhalin, C. Maguntan	6x	unknown
A213RU6x	Russia, Sakhalin, Voskhod	6x	*P. australis* Australia–East Asia
A120JP8x	Japan, Okayama	8x	*P. australis* Australia–East Asia
A136AU8x	Australia, S.A., Cortina Lake	8x	*P. australis* Australia–East Asia
A215RU8x	Russia, Sakhalin, Pokrovka, Nayba	8x	*P. australis* Australia–East Asia
A62RU10x	Russia, Sakhalin, Beregovoe	10x	*P. australis* Australia–East Asia
A133AU10x	Australia, N.S.W., Murrumbidgee River	10x	*P. australis* Australia–East Asia

^a^Lambertini *et al.* (2006).

In order to produce similar-sized, genetically identical plants for the experiment, the clones were propagated by layering of shoots horizontally in a 20–30 mm water layer in a heated greenhouse for 30 days, to initiate adventitious shoot growth at the stem nodes. When adventitious shoots were 200–300 mm tall and had developed roots, the stems were cut at both sides of the nodes and the resulting replicate plants planted in 3.5-L plastic pots (top diameter 180 mm, bottom diameter 130 mm, height 175 mm). Two shoots were planted in each pot. The pots were filled with a commercial peat soil and watered with a fertilizing solution prepared from tap water and a commercial nutrient solution (100 mg L^−1^ Pioner NPK Makro 19-2-15 + Mg and 0.1 ml L^−1^ Pioner Mikro plus with iron; Brøste, Lyngby, Denmark). In order to maintain similar water levels in all pots, each pot was placed in a black 6-L outer container (top diameter 215 mm, bottom diameter 160 mm, height 200 mm) which was filled with the fertilizing solution to a height of ∼100 mm. The plants were left to establish for 14 days and thereafter the smaller of the two plants in each pot was removed.

### Experimental set-up

A total of 150 plants were used for the experiment (75 plants received a salinity treatment, and 75 plants served as a control). Twenty days after planting, salt treatment was imposed on five replicates of each clone. The salt solution was prepared from the fertilizing solution by adding NaCl to obtain the desired salinities. The salinity treatment started at 8 ppt, and thereafter was progressively increased approximately every 14–21 days in steps of 8 ppt, to 16, 24, 32, 40, 56 and, after 120 days, 72 ppt (136, 273, 410, 547, 957 and 1230 mM, respectively). Each salinity treatment was imposed by first allowing the pots to drain for 2 h, and then flushing them five times with the new salt solution. After flushing, the outer containers were filled with the salt solution to a height of ∼100 mm. All plants were watered every second or third day to replace water lost by evapotranspiration. The plants were placed randomly on tables in a greenhouse and rotated once per week to counteract effects of climatic gradients in the greenhouse. After 14 days of exposure to each salinity treatment, plant height, *P*_max_, *g*_s_ and *E* were measured for salt-exposed as well as control plants. The controls were only measured if a minimum of two corresponding salt-exposed plants were alive. Also, dead plants were not included in the datasets.

### Environmental conditions

Air temperature, relative humidity and light conditions in the greenhouse were continuously monitored by a combined temperature and humidity sensor (Rotronic MP100TS-000, Bassersdorf, Switzerland) and a LI-190 Quantum Sensor (Li-Cor Biosciences, Lincoln, NE, USA), and all data were logged by a LI 1400 datalogger (Li-Cor Biosciences). The monthly average air temperature fluctuated from a maximum of 22 °C at noon to 14 °C at midnight in July and August, and from 20 to 11 °C in September and October. During the last 15 days of the experiment where the highest salinity level was imposed, the maximum temperature was 17.9 °C and the lowest was 10.6 °C. The average temperatures during the periods of salinity treatments fell from 22 °C during the 16 ppt treatment to 13 °C during the 72 ppt treatment. The relative air humidity fluctuated between 30 and 95 %, with strongest variations in July. Relative humidity values <50 % were rare and occurred only in July. The average humidity increased during the experiment from 61 % for the 8 ppt treatment to 82 % for the 72 ppt treatment. The average light intensity during the daytime was highest in July with a maximum of 934 µmol m^−2^ s^−1^ photosynthetically active radiation (PAR) on 26 July. Thereafter the light intensity decreased, reaching peak values of 635, 427 and 183 µmol m^−2^ s^−1^ in August, September and October, respectively. Also, the number of light hours per day decreased from 16 to 11 h during the experiment.

### Shoot elongation rate

The height of the tallest shoot in each pot was measured at the beginning and at the end of each salinity treatment as the distance from the soil surface to the apical node of the shoot. The shoot elongation rate (SER; mm day^−1^) was calculated as the difference in shoot height between two consecutive measurements divided by the number of days between the measurements. The maximum shoot height was measured as described above, when control plants had stopped increasing their shoot heights, which was after 96 days (at 56 ppt salinity).

### Gas exchange

The light-saturated rates of photosynthesis (*P*_max_), transpiration rate (*E*) and stomatal conductance (*g*_s_) were measured on the third or fourth youngest fully expanded leaf from the apex of each plant, using a LI-6400XT Portable Photosynthesis System (Li-Cor Biosciences). The leaf chamber temperature was conditioned at 20 °C and was placed on a tripod to ensure stability during readings, and supplied with atmospheric air drawn from a height of 5 m from the outside of the greenhouse. Light was supplied by a LI-6400-02B LED light source (Li-Cor Biosciences) set at an irradiance of 1800 µmol m^−2^ s^−1^ (PAR). The leaf width was measured prior to infrared gas-exchange analysis (IRGA) to estimate the leaf area in the chamber. *P*_max_, *g*_s_ and *E* were logged when the IRGA showed stable readings, usually after 2–5 min. The intrinsic water-use efficiency (iWUE) was calculated as the ratio between *P*_max_ and *g*_s_.

### Chlorophyll analyses

Two leaves (one apical and one older basal leaf) per plant were harvested, frozen and then lyophilized. Concentrations of chlorophylls (Chl *a*, Chl *b*, Chl*_a+b_*) and total carotenoids (Total-car; xanthophyll plus carotenes) were analysed by photo-spectrometry after extraction of ∼5 mg leaf dry mass (DM) in 8 mL of 96 % ethanol according to Lichtenthaler (1987). Pigment concentrations were expressed as mg g^−1^ DM, and the ratios between Chl *a* and Chl *b*, as well as the ratios between the concentration of total chlorophylls and total carotenoids [(*a* + *b*)/(*x* + *c*)], were calculated.

### Water-extractable ions

After the 56 ppt treatment, the third or fourth fully expanded leaf of the tallest shoot of each plant was harvested, frozen and lyophilized. The aboveground parts of the three surviving clones at the end of the experiment (E620CZ4x, A215RU8x and A120JP8x) were harvested and separated into the top, middle and bottom height fractions (one-third each). Each height fraction was separated into leaves and shoots, and then frozen and lyophilized. The belowground parts of the plants were washed with demineralized water and separated into rhizomes and roots. All plant samples were ground into a fine powder in a Retsch Ball Mill (Mixer Mill MM 400, Retsch, Haan, Germany). Approximately 0.1 g DM of ground plant material was extracted in 30-mL centrifugation tubes with 15 mL of Milli-Q water (Millipore) for 20 min at 80 °C. After cooling, an additional 15 mL of Milli-Q water were added and the samples centrifuged for 5 min at 1700 *g*. The concentrations of Cl^−^ in the extractions were determined by titration with a 0.0282 mol L^−1^ AgNO_3_ solution on an ABU52 Biburette Titrator (TitraMaster 85, Radiometer Analytical SAS, France). The concentrations of Na^+^, K^+^, Ca^2+^ and Mg^2+^ in the extracts were analysed by ICP-OES (Optima 2000 DV, Perkin-Elmer Instruments Inc., CT, USA). The hot-water-extractable ions largely reflect the concentrations of ions in the cytoplasm and vacuole of the cells.

### Salinity effects

As the salinity treatments were additive over time, the effects of salinity on shoot height, *P*_max_, *g*_s_, pigments and water-extractable ion concentrations in the plant tissues were statistically analysed as ratios of the measured values for each salt-treated plant to the average of the measured values for the five corresponding control plants. However, the actual measured values are shown in the figures and tables, unless stated otherwise.

For *P*_max_ and *g*_s_, the half-maximal effective concentration (EC_50_) model (Christensen *et al.* 2009) was used to estimate the concentration of salt that induced a 50 % decrease compared with the control (baseline rate). Also 20 and 80 % decreases (EC_20_ and EC_80_, respectively) were calculated with this method. The EC_50_ model assumes a Weibull distribution of the observations, and uses the experimentally derived median effective concentrations and the curve slope at the central point to estimate the EC_50_ values. Reference EC_50_ (or EC_20_ and EC_80_) with 95 % confidence limits using Weibull models were calculated by nonlinear regression on the whole dataset, using a dose–response regression program with variance weighting and proper inverse estimation (Christensen *et al.* 2009). The covariance of inhibition versus control response was taken into account for the EC confidence limit calculation.

### Statistics

Statistical analyses were performed using the software Statgraphics Centurion XV (Manugistics Inc., MD, USA). Data were tested for normal distribution and variance homogeneity using Levene's test prior to analysis and, if necessary, log-transformed to ensure homogeneity of variance. Outliers were identified by the unusual residual procedure. Values with residuals >3.5 were eliminated.

Differences among clones in the measured parameters at each salinity level were identified using one-way analysis of variance (ANOVA) with *post-hoc* Tukey's honestly significant difference (HSD) tests to identify significant differences between clones at the 95 % confidence level. A Tukey's HSD test was also used in order to identify clone-specific differences related to the inhibition of *P*_max_ and *g*_s_ (EC_20_, EC_50_ and EC_80_).

In order to compare the effects of salinity among the tested clones over the entire experiment, data for each clone were normalized by using ratios of the measured parameters of the salt-treated plants to the average of the measured parameters of the corresponding control plants (as described in the aforementioned section).

The effects of GR, PL, salinity and clonal variations were analysed by a nested ANOVA using the GLM procedure. Geographic distribution range, PL and salinity were treated as independent factors, whereas clonal variation was nested within GR × PL.

A linear regression between the highest salinity survived by each clone and the inhibition of *P*_max_ and *g*_s_ (EC_20_, EC_50_ and EC_80_) was performed.

The effects of PL and GR on the maximum shoot height, as well as on the concentrations of water-extractable ions measured in the third fully developed leaf harvested after exposure to 56 ppt salinity, were investigated. Subsequently, clonal variation within the control and the salt-exposed plant datasets was tested by one-way ANOVA.

A three-way ANOVA was used to analyse the effects of plant fraction, clone and salinity on the concentrations of water-extractable chloride and cations in the harvested plants.

A rotated factor analysis (FA) was conducted on the measured parameters of the 15 clones, to reduce the number of variables into a smaller number of principal components that account for most of the variance in the data. Since data on the concentrations of water-extractable ions were only available for one salinity level (56 ppt), while other parameters were measured at all salinities, the FA was performed in two steps. First, the average ratio values for each salinity level were calculated as explained above and an FA for data comprising physiological parameters and pigments was performed (FA1). Second, the average of the factor scores for each clone was then taken and a second Varimax rotated FA including the factor scores of FA1 and the water-extractable ion concentrations was performed (FA2).

## Results

A significant effect of salinity was measured for all parameters. However, the variation between clones was generally higher than the variation between PLs or geographic range (Table 2). The variation between the control clones was significant for all the measured parameters.

**Table 2:** Summary of a two-way ANOVA showing the effects of ploidy level (PL), geographic range (GR) and salinity on ecophysiological parameters measured in distinct *P. australis* clones. The SER, light-saturated rate of photosynthesis (*P*_max_), stomatal conductance (*g*_s_), transpiration rate (*E*), intrinsic water use efficiency (iWUE), chlorophylls (Chl *a*, Chl *b*, Chl*_a+b_*), carotenoids (Total-car), ratio of Chl *a* to Chl *b* and the ratio of Chl*_a+b_* to total carotenoids [(*a* + *b*)/(*x* + *c*)] were analysed as the ratio between salt-treated plants and the average of control plants. The factor ‘Clone’ was nested within the ‘GR × PL’ in the ANOVA. df = degrees of freedom. Values in bold indicate *P* values <0.05.

Parameter	GR (df = 1)		PL (df = 4)		Clone (GR × PL) (df = 7)		Salinity (df = 6)		Residual (df = 360–402)
	SS%	*P*	SS%	*P*	SS%	*P*	SS%	*P*	SS%
Shoot elongation rate	0.1	0.499	0.3	0.846	1.5	0.541	9.1	**0**.**000**	89.0
*P*_max_	0.0	0.731	3.2	0.169	2.5	**0**.**001**	56.2	**0**.**000**	37.9
*g*_s_	0.4	0.351	0.1	0.991	3.4	**0**.**000**	74.6	**0**.**000**	21.3
*E*	0.2	0.338	0.6	0.573	1.4	**0**.**024**	65.3	**0**.**000**	32.3
iWUE	2.3	0.114	1.4	0.771	5.6	**0**.**000**	51.9	**0**.**000**	39.5
Chl *a*	0.0	0.850	0.5	0.969	7.4	**0**.**000**	30.1	**0**.**000**	61.8
Chl *b*	0.4	0.492	0.3	0.983	6.8	**0**.**000**	26.0	**0**.**000**	66.3
Chl*_a+b_*	0.1	0.723	1.6	0.810	7.5	**0**.**000**	25.9	**0**.**000**	64.7
Chl *a/b* ratio	2.2	0.075	0.6	0.900	4.1	**0**.**001**	25.3	**0**.**000**	67.7
Total-car	3.8	**0**.**009**	1.8	0.345	2.4	0.061	22.0	**0**.**000**	69.9
[(*a* + *b*)/(*x* + *c*)]	4.6	**0**.**041**	1.8	0.711	5.8	**0**.**000**	20.8	**0**.**000**	66.8

**Figure 1.**
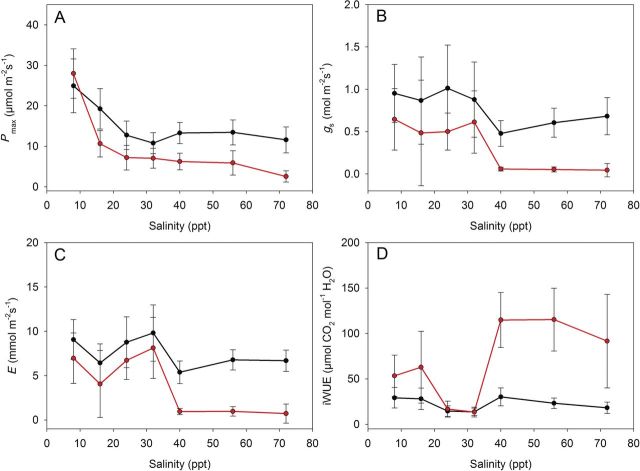
Average values of *P*_max_, *g*_s_, *E* and iWUE for the control and salt-exposed *P. australis* clones at each salinity level (8, 16, 24, 32, 40, 56 and 72 ppt). Means ± SD (*n*= 15–75). Black dots represent the control data and red dots represent the salt-treated plants.

All clones survived until 32 ppt, but some clones were already inhibited by at least 50 % in their *P*_max_ or *g*_s_ (Table 3). The first lethal effects were observed at 40 ppt, when clone A205RU4x died. Also, at this salinity, a significant negative effect of salinity was noted for all measured physiological parameters (Fig. 1A, B and C). Eleven clones survived until 56 ppt salinity, whereas only three clones (A120JP8x, A215RU8x and E620CZ4x) could cope with a salinity of 72 ppt. These three clones were the most salt-tolerant clones of the experiment.

**Table 3:** The highest salt concentration survived by the 15 distinct *P. australis* clones and the salt concentrations at which the light-saturated rate of photosynthesis (*P*_max_) and the stomatal conductance (*g*_s_) were inhibited by 20, 50 and 80 % (EC_20_, EC_50_ and EC_80_, respectively). Means ± SE are shown (*n*= 3–5); different letters within columns indicate significant differences (*P* < 0.05) between clones after Tukey's HSD test. Values in italics indicate inhibition at lower salinities and values in bold indicate inhibition at higher salinities.

Clone	Survival	*P*_max_			*g*_s_		
		EC_20_	EC_50_	EC_80_	EC_20_	EC_50_	EC_80_
E646RO4x	56 ppt	10^ns^ ± 5	30^ns^ ± 9	50^ab^ ± 11	5^ab^ ± 1	*8*^a^ ± 1	*15*^a^ ± 2
E620CZ4x	72 ppt	11^ns^ ± 6	**51**^ns^ ± 14	**75**^b^ ± 15	11^ab^ ± 5	27^bc^ ± 8	**55**^b^ ± 10
E625RO6x	56 ppt	**21**^ns^ ± 7	42^ns^ ± 6	50^ab^ ± 8	23^cd^ ± 6	**32**^c^ ± 3	42^ab^ ± 10
E656RO6x	56 ppt	18^ns^ ± 6	30^ns^ ± 8	45^ab^ ± 12	11^abc^ ± 5	28^bc^ ± 7	35^ab^ ± 7
E624RO8x	56 ppt	10^ns^ ± 5	29^ns^ ± 9	43^ab^ ± 8	11^abc^ ± 6	28^bc^ ± 10	43^b^ ± 7
E666CZ8x	56 ppt	18^ns^ ± 6	34^ns^ ± 7	54^ab^ ± 6	**28**^d^ ± 3	33^c^ ± 3	38^ab^ ± 5
E660RO12x	56 ppt	*9*^ns^ ± 5	31^ns^ ± 11	50^ab^ ± 12	*3*^a^ ± 1	12^ab^ ± 5	38^ab^ ± 8
A205RU4x	32 ppt	12^ns^ ± 5	*22*^ns^ ± 5	*35*^a^ ± 7	20^bcd^ ± 6	28^bc^ ± 4	32^ab^ ± 2
A139RU6x	56 ppt	18^ns^ ± 6	40^ns^ ± 7	56^ab^ ± 10	*3*^a^ ± 1	18^abc^ ± 9	44^b^ ± 13
A213RU6x	56 ppt	19^ns^ ± 8	40^ns^ ± 7	54^ab^ ± 12	*3*^a^ ± 1	10^ab^ ± 6	41^ab^ ± 15
A120JP8x	72 ppt	10^ns^ ± 5	43^ns^ ± 11	**76**^b^ ± 18	10^ab^ ± 3	22^abc^ ± 3	39^ab^ ± 9
A136AU8x	56 ppt	16^ns^ ± 5	34^ns^ ± 4	50^ab^ ± 7	11^abc^ ± 4	22^abc^ ± 5	39^ab^ ± 7
A215RU8x	72 ppt	10^ns^ ± 5	**51**^ns^ ± 15	**72**^b^ ± 13	7^a^ ± 3	18^abc^ ± 5	40^ab^ ± 9
A62RU10x	56 ppt	*7*^ns^ ± 3	27^ns^ ± 7	*38*^a^ ± 12	*3*^a^ ± 1	10^ab^ ± 4	43^ab^ ± 15
A133AU10x	56 ppt	*8*^ns^ ± 3	25^ns^ ± 5	50^ab^ ± 6	5^a^ ± 3	17^abc^ ± 6	36^ab^ ± 9

### Shoot height and shoot elongation

The average height of the plants at the time of planting ranged from 202 mm (A139RU6x) to 388 mm (E624RO8x), even though all clones were propagated at the same time. All control plants reached their maximum height after 96 days (at 56 ppt). At this point, their shoot height ranged between 900 mm (E666CZ8x) and 1848 mm (A133AU10x).

At 56 ppt, the salt-exposed plants were, on average, half as high as the corresponding control plants (Table 4). The most affected clone was A133AU10x (1848 mm control plant compared with 688 mm salt-treated plant; 37 % of control size) and the least affected clone was E666CZ8x (900 mm compared with 508 mm; 56 % of control size). The strongest shoot height inhibition was, however, measured for clone A205RU4x (1860 mm control versus 660 mm treatment; 35 % of control size), but this plant died after being exposed to 32 ppt salinity.

**Table 4:** Average shoot height and water-extractable ion concentrations in the third newly developed leaf of 15 distinct *P. australis* clones after exposure to 56 ppt salinity. Means are shown (*n*= 3–5) ±SE; different letters within columns indicate significant differences (Tukey's HSD *P* < 0.05) between clones (control and salt-exposed plants are tested independently).

Clone		Shoot height (mm)	Ca^2+^ (µmol g^−1^ DM)	Mg^2+^ (µmol g^−1^ DM)	K^+^ (µmol g^−1^ DM)	Na^+^ (µmol g^−1^ DM)	Cl^−^ (µmol g^−1^ DM)
E646RO4x	Control	1654^def^ ± 58	147^abc^ ± 11	74^de^ ± 5	549^cd^ ± 27	37^ab^ ± 4	410^cdef^ ± 20
	Treatment	746^de^ ± 22	122^b^ ± 11	45^ab^ ± 3	551^bcde^ ± 7	326^ab^ ± 41	567^abc^ ± 56
E620CZ4x	Control	1248^abc^ ± 62	163^abc^ ± 16	84^e^ ± 3	500^bcd^ ± 19	26^a^ ± 3	372^bcd^ ± 21
	Treatment	596^abcd^ ± 39	118^ab^ ± 9	65^bc^ ± 6	366^ab^ ± 63	435^abc^ ± 86	745^abcd^ ± 48
E625RO6x	Control	1584^cdef^ ± 34	177^bc^ ± 13	51^abcd^ ± 4	427^abc^ ± 26	71^c^ ± 6	496^efgh^ ± 31
	Treatment	682^bcde^ ± 37	74^ab^ ± 2	34^ab^ ± 2	592^cde^ ± 12	319^ab^ ± 22	707^abcd^ ± 32
E656RO6x	Control	1400^bcd^ ± 116	118^ab^ ± 10	65^cde^ ± 7	776^e^ ± 44	37^ab^ ± 2	523^gh^ ± 26
	Treatment	620^abcde^ ± 20	64^a^ ± 3	38^ab^ ± 3	574^bcde^ ± 50	349^abc^ ± 67	789^bcde^ ± 6
E624RO8x	Control	1816^ef^ ± 115	135^ab^ ± 13	54^bcd^ ± 7	422^ab^ ± 16	35^ab^ ± 4	401^cdef^ ± 19
	Treatment	796^e^ ± 30	78^ab^ ± 6	32^a^ ± 2	495^bcd^ ± 30	285^ab^ ± 30	511^ab^ ± 18
E666CZ8x	Control	908^a^ ± 52	171^abc^ ± 16	47^abc^ ± 5	588^de^ ± 40	58^bc^ ± 5	498^efgh^ ± 31
	Treatment	508^a^ ± 21	76^ab^ ± 5	30^a^ ± 2	649^cde^ ± 50	207^a^ ± 29	679^abcd^ ± 33
E660RO12x	Control	1472^bde^ ± 104	154^abc^ ± 20	59^bcde^ ± 3	553^cd^ ± 27	54^bc^ ± 6	548^h^ ± 14
	Treatment	710^cde^ ± 40	112^ab^ ± 7	54^abc^ ± 7	464^bc^ ± 67	319^ab^ ± 74	869^de^ ± 63
A139RU6x	Control	1124^ab^ ± 54	115^ab^ ± 5	29^a^ ± 2	475^bcd^ ± 50	31^a^ ± 2	302^ab^ ± 21
	Treatment	570^abc^ ± 68	99^ab^ ± 9	47^abc^ ± 3	703^de^ ± 57	221^ab^ ± 28	570^abcd^ ± 136
A213RU6x	Control	1136^ab^ ± 34	105^a^ ± 6	64^cde^ ± 5	316^a^ ± 25	31^a^ ± 2	349^bc^ ± 26
	Treatment	520^ab^ ± 23	112^ab^ ± 21	50^abc^ ± 10	487^bcd^ ± 38	693^c^ ± 114	821^bcde^ ± 112
A120JP8x	Control	1766^ef^ ± 84	169 ± 19	82^e^ ± 7	400^ab^ ± 49	28^a^ ± 2	456^defg^ ± 22
	Treatment	748^de^ ± 18	75^ab^ ± 8	45^ab^ ± 4	550^bcde^ ± 29	261^ab^ ± 30	833^cde^ ± 60
A136AU8x	Control	1340^bcd^ ± 45	115^ab^ ± 15	35^ab^ ± 2	547^cd^ ± 16	31^a^ ± 3	381^bcde^ ± 14
	Treatment	548^ab^ ± 33	80^ab^ ± 15	43^ab^ ± 6	565^bcde^ ± 32	214^a^ ± 52	532^ab^ ± 44
A215RU8x	Control	1138^ab^ ± 47	121^ab^ ± 9	43^abc^ ± 4	319^a^ ± 29	29^a^ ± 1	222^a^ ± 17
	Treatment	560^ab^ ± 12	97^ab^ ± 7	53^abc^ ± 4	178^a^ ± 5	510^bc^ ± 51	456^a^ ± 22
A62RU10x	Control	1224^ab^ ± 60	160^abc^ ± 17	57^bcde^ ± 5	446^abc^ ± 36	34^a^ ± 2	477^defg^ ± 30
	Treatment	517^a^ ± 37	97^ab^ ± 7	60^abc^ ± 6	449^bc^ ± 47	461^abc^ ± 66	1055^e^ ± 120
A133AU10x	Control	1848^f^ ± 61	204^c^ ± 8	59^bcde^ ± 5	412^ab^ ± 20	30^ab^ ± 2	392^bcde^ ± 4
	Treatment	688^bcde^ ± 24	88^ab^ ± 13	80^c^ ± 13	717^e^ ± 33	453^abc^ ± 65	735^abcd^ ± 39

**Figure 2.**
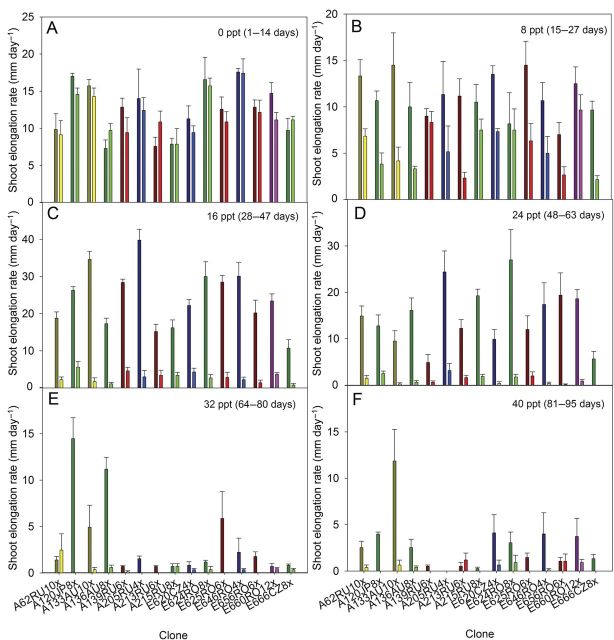
Average SER for each of the 15 *P. australis* clones at 0, 8, 16, 24, 32 and 40 ppt. For each salinity level, the corresponding number of days of growth for the control replicas is mentioned (means ± SE, *n*= 3–5). Different colours indicate different PLs, with dark tones corresponding to the control treatment and light tones corresponding to the salt treatment.

The SER of the control plants varied significantly between clones in the first 63 days of the experiment (Fig. 2). The highest SER measured was 40 mm day^−1^, after 27 days of growth (tetraploid clone from Sakhalin Island, A205RU4x).

The reduction in SER of the salt-exposed plants was significantly stronger than the time-related SER reduction of the control plants. The SERs of the salt-exposed plants varied significantly between clones only at 8 and 24 ppt. At salinities of 32 ppt and above, the SERs of stressed plants were very low (<1 mm day^−1^) for all clones and thus not significantly different (Fig. 2). The variation of the SER between clones over the entire experiment was higher than the variation between PLs or geographic origins (Table 2).

### Physiological parameters

The physiological parameters (*P*_max_, *g*_s_ and *E*) were reduced in response to increasing salinity (Fig. 1A, B and C). At the highest salinity, *g*_s_ and *E* of the salt-exposed plants were very close to zero (Fig. 1B and C). On the other hand, the iWUE of salt-exposed plants increased at high salinities (Fig. 1D).

**Figure 3.**
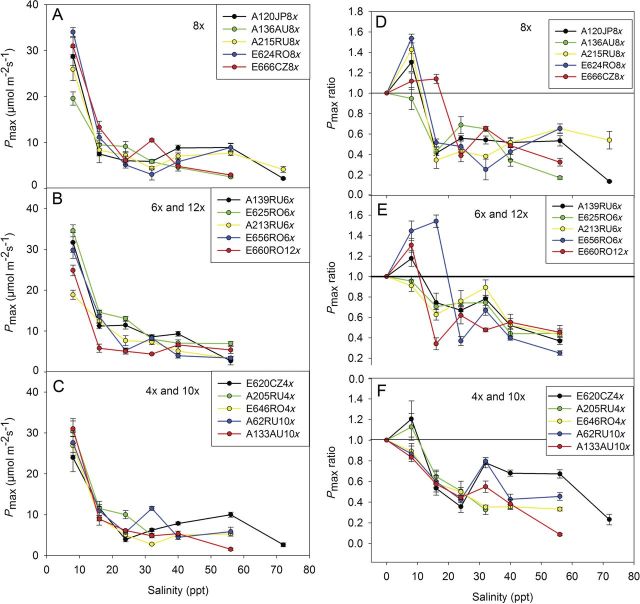
Average values of *P*_max_ of salt-exposed clones (A, B, C) and the ratio of *P*_max_ of salt-exposed *P. australis* clones to *P*_max_ of control clones (D, E, F) at each salinity level. The 15 clones are grouped according to PL (means ± SE, *n*= 3–5).

The *P*_max_ was strongly responsive to salinity. The highest *P*_max_ reduction was measured at 16 ppt (Figs 1A and 3A, B, C), when the average *P*_max_ rates fell significantly from 27.9 µmol m^−2^ s^−1^ at 8 ppt to 10.6 µmol m^−2^ s^−1^ at 16 ppt. Significant variations between clones were measured in response to salt exposure. At 16 ppt salinity, *P*_max_ ranged from 5.8 µmol m^−2^ s^−1^ (E660RO12x) to 14.6 µmol m^−2^ s^−1^ (E625RO6x). For comparison, *P*_max_ varied between 9.7 µmol m^−2^ s^−1^ (E656RO6x) and 27.0 µmol m^−2^ s^−1^ (A205RU4x) in the control plants.

The salt-induced reduction of *P*_max_ at 24 ppt salinity was smaller, but still significantly lower compared with the reduction at 16 ppt. The lowest *P*_max_ values were measured at 72 ppt (average 2.5 µmol m^−2^ s^−1^)—significantly lower than the values measured at 56 ppt (5.9 µmol m^−2^ s^−1^).

The *P*_max_ of the control clones also decreased over time. The variation ranged from an average of ∼25 µmol m^−2^ s^−1^ in July (on the figure, 8 ppt) to 13 µmol m^−2^ s^−1^ in October (after 40 ppt) (Fig. 1A). The overall average *P*_max_ during the whole experiment was 15.9 µmol m^−2^ s^−1^.

Exposure of plants to a salinity of 8 ppt had a stimulating effect on *P*_max_ for many clones (Fig. 3D, E and F). At a salinity of 16 ppt, the calculated ratio of the salt-exposed plants versus the corresponding control plants started decreasing. Nonetheless, due to the decrease in *P*_max_ for the control plants, the ratio suddenly increased at higher salinities (Fig. 3E and F).

At every salinity level, the measured values of *g*_s_ were lower for the salt-exposed plants compared with the corresponding control plants (Fig. 1B). The overall average value of *g*_s_ for the control plants was 0.78 mol m^−2^ s^−1^ (from 0.52 mol m^−2^ s^−1^ for clone E620CZ4x to 1.04 mol m^−2^ s^−1^ for clone E624RO8x). In contrast, *g*_s_ for the salt-exposed plants averaged 0.40 mol m^−2^ s^−1^ (from 0.27 mol m^−2^ s^−1^ for clone A133AU10x to 0.65 mol m^−2^ s^−1^ for clone E620CZ4x). Unlike *P*_max_, the average *g*_s_ values at salinities from 8 ppt until 32 ppt were rather constant (0.6 mol m^−2^ s^−1^). A strong inhibition of *g*_s_ was measured at a salinity of 40 ppt and higher (Fig. 1B).

The transpiration rate of salt-exposed plants did not differ significantly between 8 and 32 ppt, but was significantly reduced compared with the transpiration of control plants at 40 ppt and higher (Fig. 1C). The highest transpiration rate measured was 8.12 mmol m^−2^ s^−1^ at 32 ppt salinity (average of all salt-exposed clones; Fig. 1C). The average transpiration rate of the overall experiment for the salt-treated plants was 4.53 mmol m^−2^ s^−1^ (2.95 mmol m^−2^ s^−1^ for clone E660RO12x to 7.86 mmol m^−2^ s^−1^ for clone E624RO8x), compared with 7.60 mmol m^−2^ s^−1^ for the control plants.

The iWUE increased significantly for the salt-exposed plants at salinities of 40 ppt and higher (Fig. 1D). A maximum iWUE of 115 µmol mol^−1^ (average of all clones) was reached at 56 ppt salinity, followed by a decrease to 91 µmol mol^−1^ at 72 ppt (data for the three surviving clones). Significant differences between clones were measured. The average iWUE varied between 25 µmol mol^−1^ for clone A205RU4x and 114 µmol mol^−1^ for A215RU8x, with an overall average of 62 µmol mol^−1^ (compared with 22 µmol mol^−1^ for the control plants).

Statistical analysis of the treatment–control ratio revealed significant differences (*P* < 0.01) between clones for all measured physiological parameters. No significant effect could be assigned to either PL or GR (Table 2).

### Inhibition of *P*_max_ and *g*_s_ (EC_20_, EC_50_ and EC_80_)

**Figure 4.**
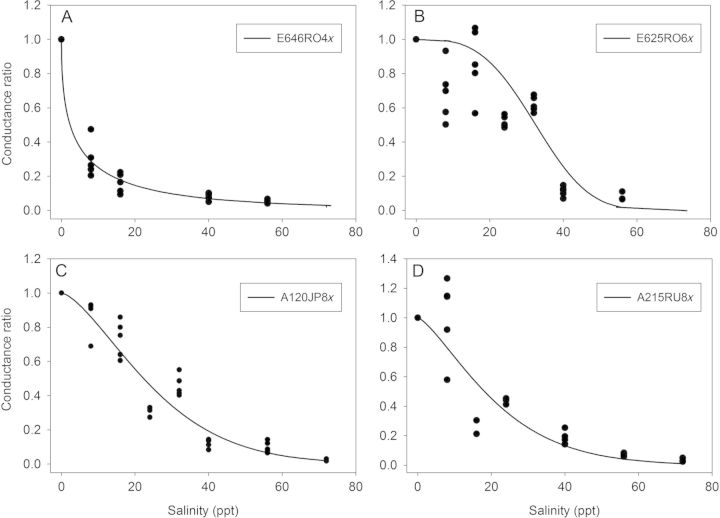
Reduction of *g*_s_ (EC_50_) in response to increasing salinity for four *P. australis* clones. 1.0 is non-inhibited and 0.0 is fully inhibited—the steepness of the curve indicates salt sensitivity (i.e. most affected clone E646RO4x, (A); moderately affected clone E625RO6x, (B); least affected A120JP8x, (C); and A215RU8x, (D)). The curves are modelled according to the Weibull distribution.

Statistical analysis of the EC_50_ values showed significant differences (*P* < 0.001) between the clones in the inhibition of *g*_s_ (Table 3) but no effects of PL or GR. Based on the statistical significance of EC_50_ for *g*_s_, salt-sensitive clones showed a strong steepness of the curve (e.g. clone E646RO4x, Fig. 4A). The *g*_s_ EC_50_ calculated for clone E625RO6x was significantly higher than the *g*_s_ EC_50_ for clone E646RO4x (Table 3; *P* < 0.001). The *g*_s_ of clone E625RO6x was stable until 20 ppt (Fig. 4B) and then strongly decreased, especially at salinities >40 ppt. The most salt-tolerant clones (e.g. A120JP8x and A215RU8x, Fig. 4C and D) had a progressive stomatal closure, in accordance with the increasing salinity.

Statistically significant differences between clones were also identified for the EC_20_ and EC_80_ of *g*_s_ (Table 3; *P* < 0.001 and 0.02, respectively). In the early-stage inhibition (EC_20_), a significant effect of GR was found (*P* = 0.03). The model indicated that the clones originating in Asia–Australia down-regulated stomatal conductance by 20 % at lower salinities, compared with the European group. The variation in *g*_s_ was, however, independent of GR at 50 and 80 % inhibition.

The calculated EC_50_ values for *P*_max_ did not differ significantly between clones. Also, no relation to PL or GR was found. However, a strong correlation (*P* = 0.003) was found between the EC_50_ of *P*_max_ and the highest salinity survived by the clones. As Table 3 shows, the 50 % inhibition, and especially 80 % inhibition (EC_80_), occurs at higher salinities for the clones that survived the longest. The EC_80_ values also differed significantly between the clones (Table 3), independent, however, of PL or GR. The three clones surviving a salinity of 72 ppt had significantly higher EC_80_ values than the most salt-sensitive clones. The correlation between EC_80_ and survival was highly significant (*P* = 0.000).

### Chlorophyll and carotenoids

All the pigment concentrations were significantly affected by salinity (Table 2). The average Chl *a* of salt-exposed plants decreased significantly from 5.2 mg g^−1^ DM (at 16 ppt) to 2.0 mg g^−1^ DM (at 32 ppt). The average Chl *b* ranged between 2.0 mg g^−1^ DM (at 16 ppt) and 0.61 mg g^−1^ DM (at 16 ppt). Similarly, Chl*_a_*_+*b*_ averaged 7.4 mg g^−1^ DM (at 16 ppt), and significantly less (2.5 mg g^−1^ DM) at 32 ppt. At the highest salinities (32–72 ppt) the differences between salinity levels were no longer significant.

The Chl *a* and Chl *b* concentrations were already generally higher in the salt-exposed plants compared with control plants after exposure to 8 ppt salinity and remained so throughout the experiment **[see** **Supporting Information****]**. The Chl*_a_*_+*b*_ average values over the entire experiment were 4.24 mg g^−1^ DM for the control plants and 4.97 mg g^−1^ DM for the salt-exposed ones.

The Chl *a*/*b* ratio and the Total-car concentration increased with increasing salinity, although significantly only until 32 and 40 ppt salinity, respectively. The Chl *a*/*b* ratio ranged between 2.56 (at 16 ppt) and 3.58 (at 32 ppt). The Total-car concentration varied between 1.31 mg g^−1^ DM (at 24 ppt) and 0.44 mg g^−1^ DM (at 40 ppt). The average [(*a* + *b*)/(*x* + *c*)] of salt-exposed plants, on the other hand, decreased from 12.3 (at 8 ppt) to 2.0 (at 32 ppt).

The variation between clones was significant not only for salt-exposed, but also for control plants **[see** **Supporting Information****]**.

The ratios between the measured values of salt-exposed plants and the average values of the corresponding control plants were significantly higher at 40 and 56 ppt for Chl*_a_*_+*b*_ and [(*a* + *b*)/(*x* + *c*)] (1.62 and 2.0, respectively), whereas the ratios of Total-car and the Chl *a*/*b* ratio decreased at the highest salinity treatments (0.91 and 0.81, respectively). The significant differences were associated with the GR only for Total-car (higher in the European clones) and [(*a* + *b*)/(*x* + *c*)] (lower in the European clones) (Table 2).

### Water-extractable ions

The concentration of water-extractable ions in the leaves harvested at 56 ppt was significantly (*P* < 0.001) different between the salt-exposed and the control plants for Ca^2+^, Na^+^ and Cl^−^, but not for Mg^2+^ and K^+^ (Table 4). The concentrations of Ca^2+^ in leaves of salt-exposed plants were 36 % lower as compared with the controls. Concentrations of Cl^−^ and Na^+^ in the salt-exposed plants (703 and 356 µmol g^−1^ DM, respectively) were 67 and 862 % higher as compared with the control plants (421 and 37 µmol g^−1^ DM, respectively). The concentration of K^+^ did not vary significantly between salt-exposed and control plants.

Values for both control and salt-exposed plants differed significantly between clones (Table 4). The Ca^2+^ concentration was lower in the leaves of salt-exposed plants, varying between 64 and 122 µmol g^−1^ DM compared with 105 to 204 µmol g^−1^ DM for the control clones. On the other hand, Cl^−^ concentrations ranged between 456 and 1055 µmol g^−1^ DM when plants were exposed to salinity, whereas Cl^−^ concentrations in the control clones varied between 222 and 548 µmol g^−1^ DM. The highest variation was measured for Na^+^. The leaves of salt-exposed plants had Na^+^ concentrations between 207 and 693 µmol g^−1^ DM, while the Na^+^ concentrations in the corresponding control plants averaged 26 to 71 µmol g^−1^ DM.

Mg^2+^ concentrations did not differ significantly between controls and salt-exposed plants, ranging from 29 to 84 µmol g^−1^ DM in the control clones, and from 30 to 80 µmol g^−1^ DM in the leaves of plants, exposed to salinity. Similarly, K^+^ concentrations varied between 316 and 776 µmol g^−1^ DM in control plants, and between 178 DM and 717 µmol g^−1^ DM in salt-exposed plants (Table 4). Again, no significant effects of PL or GR on any of the measured water-extractable ion concentrations were found (data not shown).

### Ion distribution within plant fractions

A significant effect of salinity on the concentrations of water-extractable Na^+^, Cl^−^, Ca^2+^ and Mg^2+^ (but not K^+^) was found in the plants harvested at the end of the experiment. Also, significant differences between the concentrations of all water-extractable ions in the different plant fractions (roots, rhizomes, shoots and leaves) of the three clones surviving the highest salinity (72 ppt) were found. The third-term interaction between plant fraction, clone and treatment was significant for Na^+^, K^+^, Mg^2+^ and Cl^−^, as well as the Na^+^/K^+^ ratio, but not for Ca^2+^ (Table 5).

**Table 5:** Results of a three-way ANOVA (*F*-ratios) showing the effects of clone (E620CZ4x, A215RU8x and A120JP8x), salinity (72 ppt versus control), plant fraction (roots, rhizomes, bottom shoots, middle shoots, top shoots, bottom leaves, middle leaves and top leaves) and their interactions on the water-extractable ion concentrations of the three surviving *P. australis* clones at the highest salinity of 72 ppt. df = degrees of freedom. Values in bold indicate significant *P* values: ***P < 0.001, **P < 0.01, *P < 0.05.

Ion	A: Clone (df = 2)	B: Salinity (df = 1)	C: Plant fraction (df = 7)	A × B (df = 2)	A × C (df = 14)	B × C (df = 7)	A × B × C (df = 14)
Cl^−^	7.1**	1815.7***	62.3***	3.5*	9.2***	19.1***	4.7***
Na^+^	40.8***	7242.4***	86.5***	28.2***	5.5***	9.3***	2.3**
K^+^	37.2***	0.1	32.7***	44.4***	21.0***	26.7***	3.2***
Ca^2+^	6.8***	11.7***	965.6***	6.0**	4.3***	16.7***	1.3
Mg^2+^	20.1***	24.1***	449.2***	10.9***	6.2***	23.8***	2.2**
∑ cations	16.0***	2150.1***	140.6***	15.1***	6.5***	25.3***	3.4***
Na^+^/K^+^ ratio	55.7***	4755.9***	45.2***	45.7***	14.5***	17.0***	2.6**

**Figure 5.**
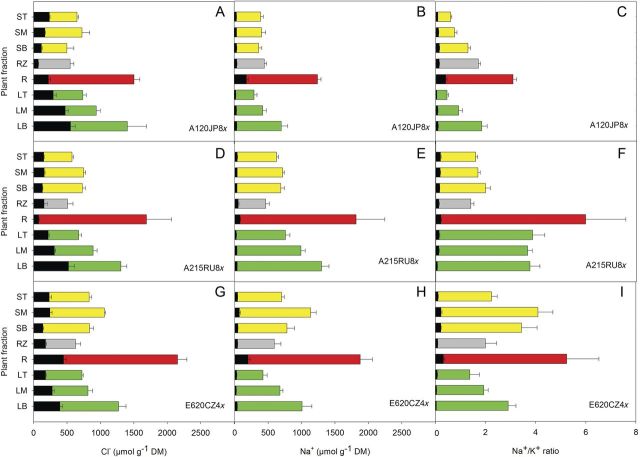
Average water-extractable Na^+^ and Cl^−^ concentrations and the Na^+^/K^+^ ratio in the different plant fractions of the three surviving clones of *P. australis* exposed to increasing salinity levels. Black bars represent the control data and coloured bars the salt-exposed plants (green for leaves, red for roots, grey for rhizomes and yellow for shoots) (means + SE, *n*= 5); LT, leaves top; LM, leaves middle; LB, leaves bottom; ST, shoots top; SM, shoots middle; SB, shoots bottom; RZ, rhizomes; R, roots.

The concentrations of Na^+^ and Cl^−^ were significantly higher in salt-exposed plants (16 times more Na^+^ and 3.8 times more Cl^−^ than in the controls) (Fig. 5). In contrast, the concentrations of Ca^2+^ and Mg^2+^ were lower in salt-exposed plants by 20 and 4 %, respectively (Table 6).

**Table 6:** Average concentrations of water-extractable K^+^, Ca^2+^ and Mg^2+^ ( µmol g^−1^ DM) in belowground parts (roots and rhizomes) and different height classes of aboveground parts (basal, middle and apical third stems and leaves) of the three surviving clones of *P. australis* at 72 ppt salinity (T) and the corresponding control plants (C). Means of five replicas are shown ±SE.

		Clone	Belowground		Stems			Leaves		
			Roots	Rhizomes	Basal	Middle	Apical	Basal	Middle	Apical
K^+^ (µmol g^−1^ DM)	C	A120JP8x	449 ± 26	267 ± 11	251 ± 12	293 ± 20	371 ± 21	410 ± 39	387 ± 29	480 ± 17
		A215RU8x	405 ± 21	456 ± 15	225 ± 6	231 ± 20	220 ± 14	616 ± 54	287 ± 33	208 ± 26
		E620CZ4x	633 ± 31	433 ± 16	214 ± 9	304 ± 23	399 ± 3	917 ± 121	517 ± 93	366 ± 10
	T	A120JP8x	405 ± 14	263 ± 16	282 ± 18	536 ± 14	656 ± 45	377 ± 17	487 ± 56	653 ± 39
		A215RU8x	312 ± 12	330 ± 12	351 ± 15	432 ± 25	397 ± 14	352 ± 20	271 ± 12	201 ± 11
		E620CZ4x	396 ± 40	307 ± 20	236 ± 15	294 ± 29	320 ± 19	354 ± 46	356 ± 15	369 ± 53
Ca^2+^ (µmol g^−1^ DM)	C	A120JP8x	44 ± 4	6 ± 0.5	12 ± 2	17 ± 0.4	33 ± 3	420 ± 47	278 ± 18	157 ± 6
		A215RU8x	43 ± 2	8 ± 0.8	10 ± 0.8	21 ± 1	37 ± 1	307 ± 16	209 ± 6	171 ± 10
		E620CZ4x	50 ± 7	8 ± 0.6	13 ± 1	20 ± 2	38 ± 2	249 ± 42	237 ± 14	169 ± 7
	T	A120JP8x	18 ± 1	6 ± 0.6	10 ± 0.9	22 ± 2	38 ± 3	289 ± 28	228 ± 35	98 ± 23
		A215RU8x	26 ± 3	5 ± 0.1	16 ± 2	28 ± 2	41 ± 1	223 ± 6	184 ± 7	131 ± 5
		E620CZ4x	31 ± 5	9 ± 1	19 ± 2	43 ± 5	48 ± 3	199 ± 19	179 ± 20	130 ± 9
Mg^2+^ (µmol g^−1^ DM)	C	A120JP8x	68 ± 4	12 ± 0.5	13 ± 1	16 ± 1	32 ± 3	241 ± 10	129 ± 9	89 ± 5
		A215RU8x	70 ± 2	20 ± 0.4	17 ± 1	22 ± 2	27 ± 2	179 ± 11	82 ± 5	65 ± 6
		E620CZ4x	79 ± 13	15 ± 1	18 ± 2	28 ± 3	37 ± 2	182 ± 56	125 ± 6	107 ± 7
	T	A120JP8x	47 ± 1	11 ± 0.4	14 ± 1	34 ± 3	44 ± 2	147 ± 18	144 ± 21	67 ± 13
		A215RU8x	69 ± 2	15 ± 0.2	33 ± 2	49 ± 5	54 ± 2	164 ± 3	134 ± 5	83 ± 3
		E620CZ4x	62 ± 4	16 ± 1	38 ± 6	64 ± 7	48 ± 5	133 ± 15	112 ± 14	83 ± 7

Different ions were located in different plant parts. Cl^−^ concentrations increased mainly in the roots (11.3 times more than control), whereas in the older leaves the relatively high Cl^−^ concentrations found in the control clones tempered the effect (Fig. 5A, D and G). In the case of Na^+^, however, the concentrations in the older leaves were 26 times higher than those in the control (Fig. 5B, E and H). Nonetheless, the roots of salt-exposed plants had the highest Na^+^ concentrations of all plant parts. The Na^+^/K^+^ ratio was very high (50 times higher than the ratio in the controls) in the older leaves and in the roots (Fig. 5C, F and I).

The salt-exposed plants had significantly higher Ca^2+^ and Mg^2+^ concentrations as compared with the control only in the middle and top shoots, as these metals were reduced in the plants in response to salt exposure. K^+^ was also accumulated predominantly in the top part of the plant (top shoots 11 % more than the control, and top leaves 45 % more than the control).

Significant differences in ion concentrations between the three surviving clones were found (Table 5). The salt-exposed replicas of clone A120JP8x had significantly lower concentrations of Na^+^ and Na^+^/K^+^ ratios in all plant parts except the rhizomes. Clone A215RU8x had the lowest root-Na^+^ concentrations in the control, yet the highest concentrations in the salt-exposed plants (Fig. 5E). The leaves of clone A120JP8x had >50 % lower Na^+^ concentration than the leaves of clone A215RU8x (476 µmol g^−1^ DM as opposed to 1016 µmol g^−1^ DM in A215RU8x). However, the leaf-Na^+^ concentration of the controls was similar between the three clones (average 31 µmol g^−1^ DM).

Clone A120JP8x had the highest concentrations of K^+^ in the salt-exposed plants, significantly higher than the K^+^ concentration in the other two clones, especially in the roots and upper stems and leaves (Table 6).

### Factor analysis

The first step of the factor analysis (FA1) that included the physiological parameters and pigments at all salinity levels extracted four main factors accounting for 86.8 % of the variation. Chlorophyll *a*, Chl *b*, Chl*_a+b_* and [(*a* + *b*)/(*x* + *c*)] had positive loadings for factor 1 (F1) and could be interpreted as a ‘pigment’-related factor. Factor 2 (F2) had high positive loadings for *g*_s_ and *E*, and negative loadings for iWUE, and could be interpreted as a ‘transpiration’-related factor. Factor 3 (F3) had positive loadings for the Chl *a*/*b* ratio and Total-car. Factor 4 had positive loading for *P*_max_ and SER and could be interpreted as a ‘growth’-related factor (F4) (Table 7).

**Table 7:** Factor analysis of the ecophysiological parameters that differed significantly among the 15 *P. australis* clones. The first step of the factor analysis (FA1) was performed for parameters measured at each of the seven salinity concentrations. The second step of the factor analysis (FA2) includes the average factor scores across salinities of the four factors in the first analysis and the concentrations of water-extractable Na^+^, Cl^−^, Ca^2+^, Mg^2+^ and K^+^ measured in the third fully developed leaf harvested at 56 ppt salinity. Variables with high component weights are shown in bold.

FA 1	Proportion of variance (%)			
	86.77			
Variables	Component weights			
	F1	F2	F3	F4
SER	0.130	0.010	0.113	**0.840**
*P*_max_	0.094	0.293	−0.005	**0.804**
*g*_s_	0.057	**0.915**	0.026	0.262
*E*	0.011	**0.944**	0.034	0.187
iWUE	0.007	**−0.873**	−0.052	0.066
Chl *a*	**0.940**	0.064	0.283	0.097
Chl *b*	**0.958**	−0.056	0.040	0.161
Chl*_a_*_+*b*_	**0.960**	0.035	0.227	0.124
Chl *a*/*b* ratio	0.093	0.350	**0.806**	0.028
Total-car	0.189	−0.152	**0.889**	0.091
(*a* + *b*)/(*x* + *c*)	**0.772**	0.070	−0.537	−0.057

**FA 2**	**Proportion of variance (%)**			
	**73.21**			
**Variables**	**Component weights**			
	**F1**	**F2**	**F3**	

Average factor scores F1	**0.846**	−0.167	0.067	
Average factor scores F2	−0.039	**0.586**	0.529	
Average factor scores F3	−0.153	**0.838**	−0.083	
Average factor scores F4	0.168	0.088	−**0.809**	
Na^+^	**0.900**	−0.093	0.005	
Cl^−^	**0.900**	0.037	−0.076	
Ca^2+^	**0.618**	−0.351	−0.163	
Mg^2+^	0.130	−**0.836**	0.160	
K^+^	0.099	−0.088	**0.927**	

The average of the factor scores for each clone across salinities was used in the second step of the Varimax rotated factor analysis (FA2) that also included the Na^+^, K^+^, Ca^2+^, Mg^2+^ and Cl^−^ concentrations. Three factors accounting for 73.8 % of the variation were extracted. Na^+^, Cl^−^, Ca^2+^ and the average factor scores of F1 (from FA1) had high positive loadings for factor 1 (F1). Factor 2 (F2) had negative loadings for Mg^2+^ and positive loadings for the average factor scores of F2 and F3 (from FA1). Factor 3 (F3) had positive loadings for K^+^ and negative loadings for the average factor scores of F4 (from FA1) (Table 7).

**Figure 6.**
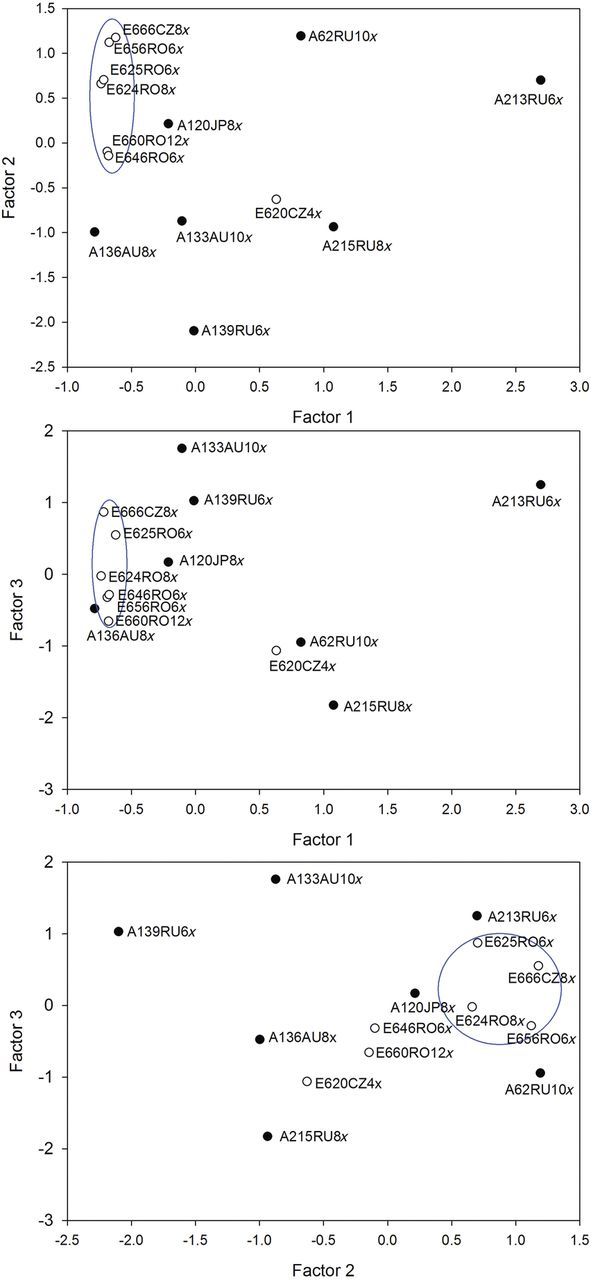
Factor score plot from a rotated FA based on all physiological parameters and water-extractable ion concentrations of the 15 distinct *P. australis* clones exposed to increasing salinity. Factor 1 is a Na^+^, Cl^−^ and Ca^2+^, as well as pigments (Chl *a*, Chl *b*, Chl*_a+b_* and [(*a* + *b*)/(*x* + *c*)]) factor, Factor 2 accounts for Mg^2+^ and physiological parameters (*g*_s_, *E* and iWUE), as well as the Chl *a*/*b* ratio and Total-car, and Factor 3 is related to K^+^, P_max_ and SER. Open symbols indicate clones from the European geographic range and black symbols indicate the Asia/Australia GR.

The FA2 revealed a grouping of the clones according to their geographic origin (Fig. 6). The positioning of the European clones in the lower range of the F1 axis reflected their lower concentrations of Na^+^ and Cl^−^. One exception is clone E620CZ4x, the one surviving at 72 ppt salinity. The distribution along the F2 axis suggests higher *g*_s_ and *E*, but also a higher concentration of Total-car in the European group, compared with several representatives of the Asia–Australia group. Nonetheless, the F3 axis isolates the two clones (E620CZ4x and A215RU8x) with the least inhibited SER and *P*_max_.

## Discussion

Our study documents the variability in salinity tolerance between 15 distinct *P. australis* clones and the possible relation to their genetic background. We investigated survival and salinity-induced inhibition of ecophysiological traits under progressively increased salinity. Owing to the design of this experiment, plants were allowed to acclimate to salt concentrations as high as 72 ppt, which would most likely be lethal when administered in a shorter experiment. The results of this study indicate significant differences between *P. australis* clones in their salinity tolerance, as well as in their specific response to salt exposure. The variation between clones is higher than the variation between PLs or phylogeographic clusters.

The main responses to salt exposure of the *P. australis* clones were: strongly inhibited SER, reduced *P*_max_ and *g*_s_, and accumulation of Na^+^ and Cl^−^ and a simultaneous reduction of Mg^2+^ and Ca^2+^ in the cells of the plant tissues, particularly the roots. Overall, these effects are similar to salinity responses reported earlier for *P. australis* (Lissner *et al.* 1999*a*; Pagter *et al*. 2009; Gorai *et al.* 2011; Zhang and Deng 2012). The clones, however, responded differently to salinity exposure. *Phragmites australis* has been reported to survive salinities of up to 500 mM (30 ppt) (Matoh *et al.* 1988). However, of the 15 clones studied in this experiment, 11 clones survived salinities up to 56 ppt and three clones survived salinities up to 72 ppt. Hence, salinity tolerance in *P. australis* is variable and depends on the genotype.

Morphological traits have previously been reported to depend on genotype by several studies (Clevering 1999; Pauca-Comanescu *et al.* 1999; Hansen *et al.* 2007). The significant variation in shoot height and SER of the control clones is in agreement with previous results (Achenbach *et al.* 2012). At 8 ppt, the SERs in salt-exposed plants were already lower than in the corresponding control plants, probably as a consequence of osmotic stress. Even though some clones (such as A120JP8x, A205RU4x) maintained a high SER at salinities up to 32 ppt, at higher salinities their SER was almost completely arrested.

Two of the surviving clones (E620CZ4x and A215RU8x) were identified in the FA as the least inhibited in their SER. Sustained SER under salt stress conditions indicates higher salinity tolerance of these clones. However, exposure to high levels of salinity unavoidably reduces the amount of energy allocated to growth, as ion exclusion and ion transport are energy-demanding processes. Furthermore, the marked reduction of the shoots' height at 56 ppt might be caused by a drop in cell expansion rate or by reduced turgor pressure (Parida and Das 2005).

The photosynthetic capacity was also affected by salinity. An unaffected or, for some clones, even higher *P*_max_ rate observed at 8 ppt salinity (Fig. 2) has been reported in previous studies (James *et al.* 2002). This might be explained by changes in cell anatomy (Munns and Tester 2008), e.g., higher chloroplast density per leaf area, as suggested by the higher chlorophyll concentrations of salt-exposed plants at 8 ppt salinity compared with the corresponding controls **[see** **Supporting information****]**. Modifications in the leaf anatomy would explain why photosynthesis, as measured per leaf area, could be sustained, although SERs were simultaneously reduced.

Not all clones investigated in this experiment were inhibited equally by salinity, suggesting that the critical salinity threshold varies between the clones. The FA indicated higher *P*_max_ for two of the surviving clones (A215RU8x and E620CZ4x), in correlation with sustained SERs and with the EC_50_ and EC_80_ models. The correlation between the inhibition of *P*_max_ (EC_50_ and EC_80_) and survival indicates the importance of maintaining photosynthesis, and thus ensuring carbon fixation, in long-term stress conditions. The model's accuracy in the upper range (EC_80_) suggests that the inhibition rate of *P*_max_ can be a good estimate in predicting a clone's salinity limit for survival.

The lower photosynthetic rate at high salinities suggests functional disturbances and possible injuries. Photosynthesis is impaired not only by closure of the stomatal, but also by the toxic effects of Na^+^ and Cl^−^ in the chloroplasts (Greenway and Munns 1980; Cuin and Shabala 2005).

Salinity is known to induce similar effects to water deficit by reducing the water potential, making the water uptake more costly (Munns 2002). To avoid water loss by transpiration, plants reduce their stomatal conductance, as observed in all the *P. australis* clones studied here. Therefore, a significant increase in the iWUE was noticed.

The differences in *g*_s_ responses identified by the inhibition model (EC_50_) indicate clone-specific stomatal adjustments (Fig. 4). The early stomatal closure of the Asia–Australia clones (Table 3, EC_20_) is supported by their positioning in the negative range of the factor 2 axis in the FA. Yet, keeping a suitable level of stomatal conductance and of transpiration is one of the trade-offs that ensure survival by allowing higher CO_2_ uptake, but also cause more salt uptake. In our results, one of the surviving clones (A120JP8x) maintained a rather high transpiration rate up to 56 ppt. Hence, this clone was physiologically better adjusted to salt exposure, which may explain its higher salinity tolerance and thus extended survival. Clone A120JP8x was also separated from the other two surviving clones on the ‘transpiration’-related factor 2 axis in the biplot (with high loadings for *g*_s_ and *E*). Furthermore, the significantly lower water-extractable Na^+^ concentrations in the different plant parts of clone A120JP8x (Fig. 6) and its positioning in the lower range of the factor 1 axis (with high loadings for Na^+^ and Cl^−^) indicated a more efficient exclusion of toxic ions for this clone.

The European clones generally grouped in the lower range of the factor 1 axis (the Na^+^ and Cl^−^ axis), reflecting the lower concentrations of ions in the leaves, compared with the Asia–Australia cluster. This suggests genetically determined differences in salt-stress responses acquired in the native range, since all clones were grown under similar conditions several years before this study and were all treated similarly. The hypothesis that the European *P. australis* is more salt-resistant than the native *P. australis* ssp. *americanus* has been raised by studies investigating the cryptic invasion of the European *P. australis* in North America (Vasquez *et al.* 2006). However, several other factors need to be considered, for example, the survival at high salinities of two Asian clones or the location of the toxic ions within the plants, as the Na^+^ concentration in the third fully developed leaf did not differ significantly between clones from the two geographic areas.

Salinity tolerance has been shown to be related to leaf ion concentrations (Munns 2002). *Phragmites australis* neither contains salt-excreting glands nor exploits the leaf abscission strategy (Lissner *et al.* 1997). We measured significant differences in the concentration of water-extractable ions, not only between clones, but also between different plant parts (Table 5). The significant differences in Na^+^ concentrations between the apical and the basal leaves of the surviving clones (Fig. 5) indicate the restriction of Na^+^ entry into the young leaves, as a protecting mechanism. Hence, the most tolerant clones were capable of sustaining high net photosynthesis rates in newly developed leaves (as high as 10 µmol m^−2^ s^−1^ at 56 ppt; Fig. 1A) despite the salt exposure and even though the older leaves were dying. Similar results have been reported by Lissner *et al.* (1997), Vasquez *et al.* (2006), Tester and Davenport (2003) and Pagter *et al.* (2009). Yet, at the highest salinity, Na^+^ reached toxic levels even in the young leaves, impairing photosynthesis (Fig. 1A). The significantly higher Na^+^ concentrations measured in the apical leaves of clone A215RU8x (Fig. 5 and factor 1 axis in Fig. 6) indicate a reduced capacity of exclusion, compared with the other two surviving clones. The very low K^+^ concentrations (factor 3 axis in Fig. 6) and the high Na^+^/K^+^ ratio (Fig. 5) indicate that K^+^ was replaced by Na^+^. Nonetheless, given the high salt concentrations to which the plants were exposed and survived, Na^+^ was most probably compartmentalized into the vacuole. Isolation of Na^+^ into the vacuole is one of the salinity tolerance mechanisms that are used in preventing ion toxicity (Zhang and Blumwald 2011).

Compartmentalization is particularly important in the roots. The proximity to the source of toxic ions and its main function of taking up water make the root extremely vulnerable to ion toxicity. Reduced K^+^ uptake due to competition from ions of similar valences on the selective root ion channels (Hu *et al.* 2005) explains the increased Na^+^/K^+^ ratio in this plant fraction. However, our results showed that some clones of *P. australis* (E620CZ4x, A120JP8x) had efficient mechanisms for excluding Na^+^ from the roots. The concentrations were, nonetheless, 10 times higher than those found in the corresponding control plants. The roots of clone A215RU8x accumulated more than twice as much Na^+^ as the other two surviving clones (Fig. 5E), suggesting vacuole isolation rather than exclusion as the main strategy for coping with salt stress. This hypothesis is further supported by the analysis of Cl^−^. Partial re-translocation of Cl^−^ ions from the leaves to the roots might be possible in the case of clone A215RU8x, since the concentrations observed in the roots of this plant are high compared with the other clones (24 times more than the concentration in controls, unlike the other two which only had 4–6 times higher concentrations than the control).

Chloride is considered less difficult to control by plants than Na^+^, because the cells' negative electric potential prevents passive uptake of Cl^−^ (White and Broadley 2001). High Cl^−^ uptake in the root (Fig. 5G) is a compensating mechanism that tries to sustain the root growth, as well as to maintain the charge balance. Nonetheless, incomplete exclusion of Cl^−^ from the leaves may negatively affect aboveground biomass production (Pagter *et al.* 2009).

The osmotic regulation of *P. australis* under salt stress may be achieved by an increased concentration of non-toxic compatible solutes, mainly K^+^ (Carden *et al.* 2003). In our study, no significant differences between the K^+^ concentrations of controls and salt-exposed plants were found. Similar results were reported by Takahashi *et al.* (2007), indicating that salt-tolerant clones of *P. australis* have efficient mechanisms for the acquisition of K^+^. The low Na^+^/K^+^ ratios in the upper parts of the shoot and especially leaves (Fig. 5) suggest equally efficient mechanisms of ion adjustment as in the above-mentioned studies. However, the up to 50 times increase in the Na^+^/K^+^ ratio in the bottom leaves of salt-exposed plants suggests significant damage to the plant organs. A key factor in expressing salt tolerance is maintaining an efficient osmotic and ionic balance, ensuring turgor and growth, and eventually survival at toxic levels. All the physiological mechanisms of the plant are relying on the efficiency of these adjustments. Therefore, salt tolerance in *P. australis* can partially be explained by (i) reduced uptake of the toxic solutes, partially achieved through efficient regulation of stomatal conductance, (ii) efficient exclusion mechanisms, potentially ensured through signalling and selective uptake, and (iii) vacuole compartmentalization of toxic ions, as for clone A215RU8x and, to a certain extent, for all clones surviving salinities >56 ppt.

The results of our study did not support our hypothesis that the salinity tolerance of *P. australis* is related to PL. However, the separation between the European and the Asia/Australia cluster in the biplots indicates that the salinity tolerance may be related to geographic origin. This is, to our knowledge, the first time that ecophysiological traits of *P. australis* clones have been shown to be correlated with their geographical origin from a longitude perspective (Europe versus Asia/Australia). The results complement the previously documented latitudinal and climatic effects (Lissner *et al.* 1999*b*; Lessmann *et al.* 2001; Bastlova *et al.* 2004). The high variation in salinity tolerance between different genotypes suggests the existence of genetically determined differences in salt tolerance mechanisms.

## Conclusions

The morphological and physiological differences between the 15 clones, as well as their distinct responses to salt stress, can most probably be attributed to the documented large genotypic variation within the species. Salt tolerance is not related to PL, but adaptations obtained at the geographic origin of the clones seem to be of importance for their salinity tolerance. The high resistance of some clones to salt stress may be important for their invasion success in some areas and for their acclimation to stress conditions. The different tolerance levels of the clones may reflect different strategies and mechanisms used for coping with salinity stress, for example, osmotic adaptation or ion exclusion. However, further studies are needed to elucidate these aspects.

## Sources of Funding

This research was funded by the Danish Council for Independent Research—Natural Sciences, via a grant to H.B.

## Contributions by the Authors

L.A. carried out the experiment and drafted the manuscript. H.B., F.E. and L.X.N. participated in the design of the study. All authors helped in drafting the manuscript and read and approved the final manuscript.

## Conflict of Interest Statement

None declared.

## Supporting Information

The following Supporting Information is available in the online version of this article –

**File 1.** Table. Average pigment concentrations (Chl*_a_*, Chl*_b_*, Chl*_a_*_+*b*_ and Total-car) as well as the Chl *a*/*b* ratio and the [(*a* + *b*)/(*x* + *c*)] ratio for the 15 *P. australis* clones exposed to 8, 16, 24, 32, 40, 56 and 72 ppt salinity. Different letters indicate significant differences between the clones (Tukey's HSD, *P* < 0.05).

Additional Information
