# Supplementary material for: Differences in salinity tolerance of genetically distinct Phragmites australis clones
Source: AoB Plants. 2013 Mar 11;5:plt019. doi: 10.1093/aobpla/plt019 (PMC4104622; doi:10.1093/aobpla/plt019)
Supplement: Additional Information [file supp_5_plt019_index.html]

Differences in salinity tolerance of genetically distinct Phragmites australis clones — Additional Information 

# Differences in salinity tolerance of genetically distinct *Phragmites australis* clones

## Additional Information

**Files in this Data Supplement:**

- Additional Information - Additional Information
